# Factors associated with adherence to Antiretroviral Therapy (ART) among adult people living with HIV and attending their clinical care, Eastern Ethiopia

**DOI:** 10.1186/s12914-015-0071-x

**Published:** 2015-12-28

**Authors:** Shiferaw Letta, Asrat Demissie, Lemessa Oljira, Yadeta Dessie

**Affiliations:** Haramaya University, College of Health and Medical Sciences, School of Nursing and Mid wifery, Harar, Ethiopia; Addis Ababa University, School of Nursing and Midwifery, Addis Ababa, Ethiopia; Haramaya University, College of Health and Medical Sciences, Department of Public Health, Harar, Ethiopia

**Keywords:** ART, Adherence, HIV/AIDS, Clinical care, Depression, Disclosure status

## Abstract

**Background:**

To attain a successful treatment outcome, Antiretroviral Therapy (ART) treatment for people living with HIV requires more than 95 % adherence level. The adherence level varies depending on different population contexts. Thus, the objective of this study was to investigate ART adherence level among HIV positive patients attending their clinical care in public health facilities in Harar and Dire Dawa, Eastern Ethiopia.

**Methods:**

We conducted a cross-sectional study among 626 ART attendees. Data were collected using a structured questionnaire with a face-to-face interview. ART adherence was considered when taking all antiretroviral treatment in a correctly prescribed doses at a right time (no dose missed or delayed for greater than or equal to 90 min) in the week prior to the study. Multivariable logistic analysis was applied to examine the association between the dependent and independent variables. Statistical significance was set at *p*-value <0.05.

**Results:**

The level of ART adherence was 85 %. Adherence was more likely among patients of 35–44 years (AOR = 2.39; 95 % CI = 1.15–5.01), had monthly income of 501.00–999.00 Ethiopian Birr (ETB) (AOR = 6.73; 95 % CI = 2.71–16.75), no history of opportunistic infection (AOR = 2.81; 95 % CI = 1.47–5.36), and had good family support (AOR = 2.61; 95 % CI = 1.45–4.72). However, those who did not disclose their sero-status (AOR = 0.45; 95 % CI = 0.21–0.97) and did experience depression (AOR = 0.36; 95 % CI = 0.21–0.61) were less likely adherent than their counter parts.

**Conclusions:**

The level of ART adherence was sub-optimal. Concerted and collaborative efforts through effective and efficient interventions are needed in view of the identified factors in order to improve the adherence level.

## Background

The increased availability of ART has essentially improved the survival rates through lowering incidence of opportunistic infections among people living with HIV. Even though access to ART is vital; ensuring the patients’ adherence to the prescribed regimen is equally important. In order to attain a successful treatment outcome, the current treatment for HIV/AIDS regimen requires adherence level of greater than 95 % [1–5]. 

Improving adherence requires collaboration with the patient in an effort to understand and improve individual impediments to adherence. This can generally be done by establishing dedicated time to educate every patient, plan for adherence, and maintain support and collaboration throughout the course of treatments. In this way, adherence can regularly be assessed, problems can be averted, and side effects can be dealt [6]. There are many alterable factors known to affect the treatment adherence. These are depression, regimen complexity, medication side effects, and relationship between patients and care providers which should be addressed prior to starting their treatments and throughout the ongoing treatments [7–9].

In Ethiopia, a numbers of studies have been conducted on ART adherence and factors associated with ART adherence [7–10]. The studies have reported a range of factors influencing ART adherence at various levels; however, the findings were varying depending on the contexts of the studies. In this regard, there have been very limited researches in Eastern Ethiopia. Therefore, with this study, we intended to determine the adherence level and its associated factors among adult people living with HIV and attending their clinical care in public health facilities found in Harar and Dire Dawa towns, Eastern Ethiopia.

## Methods and procedures

### Study setting and participants

The study was conducted in Harar and Dire Dawa towns which are located in the East of Addis Ababa—the capital city of Ethiopia. There were seven health centers and one public health hospital in Dire Dawa town, and three health centers, two military and two public hospitals in the Harar town which were providing ART service and care for patients [11].

People living with HIV/AIDS, who are 18 years old and above, able to hear and communicate well or mentally fit and being on ART at least for 3 months were included in the study. The sample size of the study was calculated using a single population proportion formula with the assumptions of 95 % Confidence Level (CL), marginal error (d) of 0.05, and adherence level (P) of 0.74 % which was taken from previous study in Ethiopia [10]. Through applying a finite population correction, the initial calculated sample size was 296. After adding 10 % non-response rate and considering a design effect of two, the final sample size calculated was 626**.** A multi stage sampling technique was applied to select study participants. First, from seven health centers and one public health hospital in Dire Dawa, three health centers and one hospital were randomly selected. In the same manner, in Harar, one from the two hospitals and two from the three health centers were selected. The calculated sample size was allocated in proportional to the number of patients in each health facility. Finally, the respondents were selected through simple random sampling technique by using a sampling frame that was developed from the registration book of the patients.

### Data collection

Face-to-face interview was applied to collect the data. Seven trained Diploma and Bachelor of Science (BSc) holder nurses who can speak the local languages fluently: Afan Oromo, Amharic and Semoligna collected the data. The interviews were undertaken in private room after signed written consent from the study subjects. Pretest was done on 5 % of the total sample size at Chiro Hospital which is one of the government hospital not included in the study. Interviewers and supervisors were trained for 3 days prior to the implementation of the actual field work on the objective, data collection techniques, maintaining data quality, and techniques of interview of the study. The data consistency and completeness were checked on daily basis by supervisors and the principal investigator.

### Measurements

ART adherence was defined as taking all pills in correctly prescribed doses at right time (no dose missed or delayed for greater than or equal to 90 min) [7]**.** According to this study, the adherence was measured by self- reported 7 day recall dose adherence. A structured pre tested questionnaire which was developed from different literatures was used for the purpose of data collection. The dependent variable of the study was adherence to ART and the independent variables were socio-demographic and economic factors, psychosocial and medication related characteristics. Depression was measured using a scale of 20-items from Center for Epidemiological Studies Depression which helps to examine different manifestations; for instance, restlessness, sleeplessness, poor appetite, loss of interest and feeling of loneliness in the week right before the study [12]. The internal consistency (Cronbach Alpha) of the scale was greater than 0.80. The responses were summed and those scored above the mean were categorized as depressed.

### Data analysis

The data was cleaned, coded and entered in to Epidata software (Version 3.1) and exported to SPSS version 16.0 for analysis. First the descriptive analysis was conducted and the result was displayed in the form of tables, charts and figures. Then binary logistic analysis was conducted to measure the association between the dependent variable and independent variables: patient factors (socio-demographic, economic, socio cultural and psychosocial factors), medication and related characteristics (pill burden, treatment complexity, missed dose and medication side effects), system factors include access and health care providers’ related characteristics and social support, stigma, and disclosure status were among community related factors using odds ratio and 95 % confidence interval. Finally, multivariable logistic regression analysis was conducted in order to identify the factors associated with the adherence level. Statistical significance was set at *P*-value <0.05.

### Ethical considerations

Ethical approval for the study was secured from Institutional Research Ethics Review Committee of Haramaya University College of Health and Medical Sciences. Important information about the purpose of the study and its procedures were explained for the respondents with the assurance of maintaining their confidentiality in a strict manner. Participation in the study was based on each patient’s will and ability to give informed consent. Detail explanations had been given and they were assured that disagreements and discontinuations from the study did not have a negative effect on services to be provided at any time. The interviews were made after getting written informed consents from each patient.

## Results

### Brief participants’ characteristics

A total of 620 adult HIV patients on ART participated in the study. The mean age (±SD) of study participants was 36.7 (±10.7) where 42.3 % were in age group of 25–34 years; male constituted 313 (50.5 %); most of the study participants 254 (41 %) were married; and 287 (46.0 %) of them had attended elementary school. About one-third, 161 (26 %) were daily laborers; 133 (22 %) earned <500.00 Ethiopian Birr (ETB) (20.00 ETB = 1USD) per month; and 322 (56.8 %) had 2–4 family members (Table 1).

**Table 1:** Socio-demographic and economic characteristics among adult patients on ART in public health institutions in Harar and Dire Dawa towns, Eastern Ethiopia, 2012

Characteristics	Frequency(*N* = 620)	Percent (%)
Age in years		
18–24	41	6.6
25–34	262	42.3
35–44	177	28.5
>=45	140	22.6
Sex		
Male	313	50.5
Female	307	49.5
Religion		
Orthodox	320	51.6
Muslim	201	32.4
Protestant	74	11.9
Others	25	4.0
Marital status		
Married	254	41.0
Single	98	15.8
Divorce/separated	148	23.9
Widowed	120	19.4
Level of education		
Unable to read and write	106	17.1
1–8	287	46.0
9–12	160	25.8
12+	67	10.8
Occupation		
Government employee	59	9.5
Private employee	112	18.1
Daily laborer	117	18.9
Merchant	50	8.1
Have no job	182	29.4
Others	134	16.0
Average monthly income		
<500.00 ETB	130	21.0
501.00–999.00ETB	66	10.6
>1000.00 ETB	75	12.1
Difficult to determine	349	56.3

### Medication and related characteristics

The majority of the respondents, 420 (67.7 %), were tested for HIV before 24 weeks (2-years) and among those patients who were on ART, 369 (59.5 %) had started ART before 24 weeks (2 years). Based on patients’ record review, the respondents were on combination of ART drugs with regimen of Zidovudine (AZT), Lamivudine (3TC), Nevirapine (NVP), Efavirenz (EFV), Stavudine (d4t) and Tenofovir (TDF). About one-third of the participants 189 (30.1 %) were on AZT-3TC-NVP. High proportion of patients 293 (47.3 %) were taking 3 tablets per day while few of the respondents 13 (2.1 %) were taking more than or equal to 5 tablets per day including non-ART drugs (Table 2).

**Table 2:** Medication and related characteristics of adult patients on ART in public health institutions in Harar and Dire Dawa towns, Eastern Ethiopia, 2012

Characteristics	Frequency(*N* = 620)	Percent (%)
Duration after knowing sero status in weeks		
3–12 weeks	138	22.30
13–24 weeks	62	10.00
>24 weeks	420	67.70
Duration after ART initiation in weeks		
1–12 weeks	189	30.50
13–24 weeks	62	10.00
>24 weeks	369	59.50
ARV drug used		
AZT-3TC-EFV	116	18.7
AZT-3TC-NVP	189	30.10
D4T-3TC-EFV	64	10.30
D4T-3TC-NVP	64	10.30
Others	187	30.20
Experienced side effects		
Yes	232	37.40
No	388	62.60
Drugs other than ARV		
Yes	462	74.50
No	158	25.50
Number of pills taken in a day		
2 tablets	261	42.10
3 tablets	293	47.30
4 tablets	53	8.50
>=5 tablets	13	2.10
History of opportunistic infections(OIs)		
Yes	222	35.80
No	398	64.20
Doses of ART status in the last 7 days		
Missed	93	15.00
Not missed	527	85.00
Number of doses missed in last 7 days		
1–2 doses	64	69.00
3–4 doses	13	14.00
5–7 doses	16	17.00

### ART adherence level and reasons for missing their treatment

**Fig. 1:**
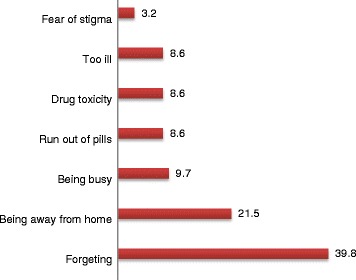
Reasons for skipping ARV drugs among adult patients on ART in public health institutions in Harar and Dire Dawa towns, Eastern Ethiopia, 2012

The level of dose adherence was 85 %; whereas, the rest 15 % missed doses (one and more) of their drugs in the last seven days (non-adherent). The main reasons given for missing their treatment were forgetting 37(39.8 %), being away from home 20 (21.5 %), and followed by being busy with different activities (Fig. 1).

### Factors associated adherence to ART

Patients who were in the age of 35–44 years old were 2.4 times (AOR = 2.39; 95 % CI = 1.15–5.01) more adherent than those older age group (≥45 years). Patients who earned an average income of 501–999 ETB per month were 6.73 times (AOR = 6.73; 95 % CI = 2.71–16.75) more likely adhered than those earned less than 500.00 ETB. Patients who took two tablets (AOR = 12.98; 95 % CI = 2.78–60.59), three tablets (AOR = 12.90; 95 % CI = 2.87–57.94) and four tablets (AOR = 5.87; 95 % CI = 1.02–28.54) per day were more adherent than those taking five and more tablets. Those who did not have history of opportunistic infection were 2.8 times (AOR = 2.81; 95 % CI = 1.47–5.36) more likely adhered than those experienced opportunistic infection. The odds of adhering to ART was 2.6 times higher among those getting good family support (AOR = 2.61; 95 % CI = 1.45–4.72) than those who had poor family support. On the other hand, adherence was less by 55 % (AOR = 0.45; 95 % CI = 0.21–0.97) among patients who did not disclose their sero-status to any family member than their counter parts and by 64 % (AOR = 0.36; 95 % CI = 0.21–0.61) among patients who did experience depression compared to those who did not (Table 3).

**Table 3:** Factors associate with ART adherence among adult patients on ART in public health institutions in Harar and Dire Dawa towns, Eastern Ethiopia, 2012

Characteristics	Adherence status	COR(95 % CI)	AOR(95 % CI)	
	Adhered *n* (%)	Not adhered *n* (%)		
Age in years				
18–24	32 (78.0)	9 (22.0)	0.97 (0.42–2.25)	0.84 (0.31–2.25)
25–34	226 (86.3)	36 (13.7)	1.71 (1.00–2.29)	1.27 (0.68–2.37)
35–44	159 (89.8)	18 (10.2)	2.41(1.28–4.54)	2.40 (1.15–5.01)*
>=45	110 (78.6)	30 (21.4)	1	1
Average income/month				
<500.00ETB	123 (94.6)	7 (5.4)	1	1
501–999.00 ETB	61 (92.4)	5 (7.6)	4.09 (1.828–9.187)	6.73 (2.71–16.75)*
>1000.00 ETB	60 (80.0)	15 (20.0)	2.85 (1.100–7.359)	1.62 (0.57–4.58)
Not determined	283 (81.1)	66 (18.9)	0.93 (0.99–1.745)	1.21 (0.59–2.49)
Waiting time				
≤30 min	364 (87.5)	52 (12.5)	1.70 (1.12–2.76)	1.36 (0.79–2.34)
>30 min	163 (79.9)	41 (20.1)	1	
Depression				
Yes	257 (81.3)	59 (18.7)	0.55 (0.35–0.87)	0.36 (0.213–0.614)*
No	270 (88.8)	34 (11.2)	1	1
Pill burden				
2 tablets	221 (84.7)	40 (15.3)	3.45 (1.08–11.09)	12.98 (2.781–60.59)*
3 tablets	257 (87.7)	36 (12.3)	4.46 (1.38–14.38)	12.90 (2.87–57.94)*
4 tablets	41 (77.4)	12 (22.6)	2.14 (0.59–7.75)	5.87 (1.21–28.54)*
5 tablets and more	8 (61.5)	5 (38.5)	1	1
Substance use				
Used	158 (77.8)	45 (22.2)	0.46 (0.29–0.71)	0.612 (0.37–1.03)
Not-used	369 (88.5)	48 (11.5)	1	
Opportunistic infections(OIs)				
Not encountered	198 (89.2)	24 (10.8)	1.73 (1.05–2.24)	2.81 (1.47–5.36)*
Encountered	329 (82.7)	69 (17.3)	1	1
Disclosure status				
No	402 (83.4)	80 (16.6)	0.52 (0.28–0.97)	0.45(0.21–0.97)*
Yes	125 (90.6)	13 (9.4)	1	1
Family support				
Good	232 (89.2)	28 (108)	1.83 (1.14–2.94)	2.61(1.47–4.72)*
Poor	295 (89.1)	65 (18.1)	1	1
Adherence counseling				
Yes	509 (85.8)	84 (14.2)	3.03 (1.32–6.69)	2.45(0.37–1.03)
No	18 (66.7)	9 (33.3)	1	
Well-skilled counselor				
Yes	497 (86.1)	80 (13.9)	3.27 (1.47–7.29)	1.22(0.311–4.79)
No	30 (69.8)	13 (30.2)	1	
Satisfaction to counselor				
Satisfied	493 (89.2)	81 (14.1)	2.15 (1.07–4.32)	1.24(0.38–4.08)
Not satisfied	34 (73.9)	12 (26.1)	1	

*Statistically significant association (*P* <0.05)

## Discussion

The level of adherence identified was 85 %. The reasons for skipping doses were forgetting to take, being far from home, being busy and running out of pill. Factors associated with the ART adherence were age, income, pill burden, opportunistic infections, disclosure and depression status and family support.

The level of adherence identified here was 85 % which is lower than the recommended level of adherence. In the current recommendation, at least 95 % of ART adherence level is required to suppress viral replication, show clinical improvement and increased CD4 count [13]. This implicates maximum effort still are needed to push up the adherence to the status of the recommended level. Despite the adherence level is less than the recommended level; the obtained result was higher than other studies in different parts of Ethiopia such as Addis Ababa [10], Yirgalam [6], and Jimma [7, 8]. One possible explanation might be many of the participants in this study have been on ART for longer duration, and those taking the drug/s for a longer duration usually acquire skills how to deal with some of the obstacles hindering them not to adhere. Further, context variations may also explain the difference observed.

The main reasons for skipping were forgetting to take, being far from home, being busy, running out of pill, drug toxicity, too ill and fear of stigma which were similar to other studies in Jimma [7, 8], Nigeria [14, 15], Kenya [8, 9, 14, 16, 17]. The reported reasons implicate a strong message for future intervention that can be tailored to each of them. For example, for the reason mentioned forgetting, it is possible to craft an innovative way like reminding them through message using mobile technology. At the same time, for patients who claimed being away from home as a reason, it is possible to encourage them plan a kind of regular reminder for themselves every time the patient going away from their residential home.

The level of adherence was significantly influenced by age that patients in age group of 35–44 years old were more likely to be adherent than the younger and older. This is in agreement with a study conducted in Kenya that had found adherence to ART increased with increased age and decrease as the age goes beyond 60 years [14]. The possible explanation of this might be the younger might fear sigma and discrimination compared with middle aged adults. On the other hand, when they are getting older, the cognitive challenges can exist so that it decreases the adherence to the treatment.

Patients who had an average income of 501–999.00 ETB were more likely adhered than those earned less average monthly income (<500.00 ETB). However, a study in Kenya found that employed patients who had higher monthly income were less adhered to treatments [14]. The explanation of this may indicates that lower level income can expose them to various psychological issues which can hamper the adherence. It is also apparent that patients who have higher level income are usually those employed and engaged in business works which in one way can subject them busy and away from home periodically.

Depressed patients were about 0.36 times less likely adherent than those non-depressed. Other comparable findings were also documented from within and outside of the country [8–10, 15]. This might be explained as that those depressed patients usually experience hopelessness and demoralization which can expose them skipping or forgetting their regular treatment. This finding has a strong implication that there is a need to design to screen patients on a regular bases and then provide them appropriate counseling.

Those who did not disclose about own sero-status was found to be associated with adherence similar to other studies conducted in different parts of Ethiopia [8, 10]. When there is no self- disclosure, the persons may fear to take their treatments when other people present. In such case there is a need to develop skill to maintain adherence in the non-disclosure state or design a way to encourage for disclosure.

Those patients taking less pill burden were more likely adherent. The finding is comparable with previous reports that pill burden likely decreases the ART adherence [18, 19]. This might be associated with when a number of pill increases, it may subjects them to experience more adverse effects from the medications which potentially lead them to skip their treatment.

It was revealed that patients who did not encounter opportunistic infections had more adherences to their treatment which is consistent with the finding of other study in, United State of America [21] and England [22]. This might show the multiple occurrences of the infections potentially increase pill burden which can be associated with increased level of adverse reaction so that influence the treatment adherence.

Patients who got family support were more likely adhered than the counterparts consistent with studies in Jimma [8, 9]. Most of the time, the support start from accepting HIV result that would result in disclosing their status possibly received support from their family and friends which have immediate and long term positive influences on their adherence [20].

The finding of this study should need to be interpreted with some limitations. The measurement of adherence was only relied on patients’ report of missed doses. It may be subjected to social desirability and recall bias which leads to overestimation of adherence level.

## Conclusions

The ART adherence level in this study was 85 % which is sub optimal (<95 % adherence level). It was revealed that the patient age, middle level of monthly income, depression, pill burden, opportunistic infections, disclosure status and family support were the predictors of ART adherence. Collaborative efforts of the Regional Health Office, Regional HIV/AIDS prevention coordinating office, adherence counselors and adherence supports were recommended to draw effective and efficient interventions targeting these factors.

## Abbreviations

AIDS: Acquired immune deficiency syndrome
ART: AntiretroViral therapy
ARV: Anti retro viral
AZT: Zidovudine
CD4: Cluster differentiation
CSA: Central statistical authority
D4t: Stavudine
DACA: Drug administration and control authority
EDHS: Ethiopian demographic and health survey
ETB: Ethiopian birr
FDC: Fixed dose combination
FMOH: Federal ministry of health
HIV: Human immune deficiency virus
MOH: Ministry of health
NRTI: Nucleoside reverse transcriptase inhibitors
NVP: Nevirapine
PLWH: People living with HIV
RHAPCO: Regional HIV/AIDS prevention and coordinator office
SNNPR: South nation nationalities and peoples region
WHO: World health organization
