# Erratum to: Factors associated with adherence to Antiretroviral Therapy (ART) among adult people living with HIV and attending their clinical care, Eastern Ethiopia

**DOI:** 10.1186/s12914-016-0078-y

**Published:** 2016-03-02

**Authors:** Shiferaw Letta, Asrat Demissie, Lemessa Oljira, Yadeta Dessie

**Affiliations:** Haramaya University, College of Health and Medical Sciences, School of Nursing and Midwifery, Harar, Ethiopia; Addis Ababa University, School of Nursing and Midwifery, Addis Ababa, Ethiopia; Haramaya University, College of Health and Medical Sciences, Department of Public Health, Harar, Ethiopia

It has come to the publisher’s attention that in the original article [1], Table 3 was incorrectly formatted. The correct formatting of the table has been updated in the original article, and published in this Erratum for quick reference, see Table 1.

**Table 1:** Factors associate with ART adherence among adult patients on ART in public health institutions in Harar and Dire Dawa towns, Eastern Ethiopia, 2012

	Adherence status			
Characteristics	Adhered No (%)	Not Adhered No (%)	COR (95%CI)	AOR (95%CI)
Age in years				
18–24	32 (78.8)	9 (22.0)	0.97 (0.42–2.25)	0.84 (0.31–2.25)
25–34	226 (86.3)	36 (13.7)	1.71 (1.00–2.29)	1.27 (0.68–2.37)
35–44	159 (89.9)	18 (10.2)	2.41 (1.28–4.54)	2.40 (1.15–5.01)*
> = 45	110 (78.6)	30 (21.4)	1	1
Average income/month				
< 500.00ETB	123 (94.6)	7 (5.4)	1	1
501–999.00 ETB	61 (92.4)	5 (7.6)	4.09 (1.828–9.187)	6.73 (2.71–16.75)*
> 1000.00 ETB	60 (80.0)	15 (20.0)	2.85 (1.100–7.359)	1.62 (0.57–4.58)
Not determined	283 (81.1)	66 (18.9)	0.93 (0.99–1.745)	1.21 (0.59–2.49)
Waiting time				
≤ 30 min	364 (87.5)	52 (12.5)	1.70 (1.12–2.76)	1.36 (0.79–2.34)
< 30 min	163 (79.9)	41 (20.1)	1	
Depressiom				
Yes	257 (81.3)	59 (18.7)	0.55 (0.35–0.87)	0.36 (0.213–0.614)*
No	270 (88.8)	34 (11.2)	1	1
Pill burden				
2 tablets	221 (84.7)	40 (15.3)	3.45 (1.08–11.09)	12.98 (2.781–60.59)*
3 tablets	257 (87.7)	36 (12.3)	4.46 (1.38–14.38)	12.90 (2.87–57.94)*
4 tablets	41 (77.4)	12 (22.6)	2.4 (0.59–7.75)	5.87 (1.21–28.54)*
5 tablets and more	8 (61.5)	5 (38.5)	1	1
Substance use				
Used	158 (77.8)	45 (22.2)	0.46 (0.29–0.71)	0.612 (0.37–1.03)
Not-used	369 (88.5)	48 (11.5)	1	
Opportunistic infections(Ols)				
Not encountered	198 (89.2)	24 (10.8)	1.73 (1.05–2.24)	2.81 (1.47–5.36)*
Encountered	324 (82.7)	69 (17.3)	1	1
Disclosure status				
No	402 (83.4)	80 (16.6)	0.52 (0.28–0.97)	0.45 (0.21–0.97)*
Yes	125 (90.6)	13 (9.4)	1	1
Family support				
Good	232 (89.2)	28 (108)	1.83 (1.14–2.94)	2.61 (1.47–4.72)*
Poor	295 (89.1)	65 (18.1)	1	1
Adhernce counseling				
Yes	509 (85.8)	84 (14.2)	3.03 (1.32–6.69)	2.45 (0.37–1.03)
No	18 (66.7)	9 (33.3)	1	
Well-skilled counselor				
Yes	497 (86.1)	80 (13.9)	3.27 (1.47–7.27)	1.22 (0.311–4.79)
Satisfaction to counselor				
Satisfied	493 (89.2)	81 (14.1)	2.15 (1.07–4.32)	1.24 (0.38–4.08)
Not satisfied	34 (73.9)	12 (26.1)	1	

*Statistically significant association (*P* <0.05)

In addition, the authors noticed errors in certain reference citations within the discussion section following the publication of [1]. The section of text affected has now been amended in the original article [1], and a list of the changes made has been listed for quick reference:

The correct reference for ‘Yirgalam’ is: Markos E, Worku A, Davey G. Adherence to ART in PLWHA at Yirgalem hospital, South Ethiopia. Ethiop J Health Dev. 2008;22(2):174–9.

The correct references for ‘Jimma’ are: Amberbir A, Woldemichael K, Getachew S, Girma B, Deribe K. Predictors of adherence to antiretroviral therapy among HIV-infected persons: a prospective study in Southwest Ethiopia. BMC Public Health. 2008;8:265; and Tiyou A, Belachew T, Alemseged F, Biadgilign S. Predictors of adherence to antiretroviral therapy among people living with HIV/AIDS in resource-limited setting of southwest Ethiopia. AIDS Res Ther. 2010;7:39.

The correct references for ‘Nigeria’ are: Bello SI. HIV/AIDS patients’ adherence to Antiretroviral therapy in Sobi specialist hospital, Ilorin Nigeria. J Adv Sci Res. 2011;2(3):52–7; and Erah P, Arute J. Adherence of HIV/AIDS patients to antiretroviral therapy in a tertiary health facility in Benin City, Nigeria. Afr J Pharm Pharmacol. 2007;2(7):145–52.

The correct reference for ‘Kenya’ is: Anthony N. Factors that influence non-adherence to antiretroviral therapy among HIV and AIDs patients in central province, Kenya, 2011. http://ir-library.ku.ac.ke/handle/123456789/1725.
